# Examining the relationship between the food environment and adult diabetes prevalence by county economic and racial composition: an ecological study

**DOI:** 10.1186/s12889-017-4658-0

**Published:** 2017-08-09

**Authors:** Lindsey Haynes-Maslow, Lucia A. Leone

**Affiliations:** 1 Department of Agricultural and Human Sciences, School of Public Health and Health Professions North Carolina State University, 512 Brickhaven Drive, Suite 240, Campus Box 7606, Raleigh, NC 27695 USA; 2 Department of Community Health and Health Behavior, School of Public Health and Health Professions, University at Buffalo, Buffalo, NY USA

**Keywords:** Food environment, Diabetes, Food access, Health disparities

## Abstract

**Background:**

Inequitable access to healthy food may contribute to health disparities. This study examines the relationship between the prevalence of adult diabetes and food access in the U.S. by county economic/racial composition.

**Methods:**

An ecological study from 2012 was used to estimate the relationship between diabetes and retail food outlet access. County diabetes prevalence was measured based on individual responses to the Behavioral Risk Factor Surveillance Survey question, “Have you ever been told by a doctor that you have diabetes?” If the answer was “yes” individuals were classified as having diabetes. Retail food outlets included grocery stores, supercenters, farmer’s markets, full-service restaurants, fast food restaurants and convenience stores. Counties were categorized as “high-poverty” or “low-poverty”. Counties were categorized as low (< 4.6%), medium (4.6%–31.0%), and high (> 31.0%) percent minority residents. Multiple linear regression models estimated the association between retail food outlets and diabetes, controlling for confounders, and testing for interactions between retail food outlets and county racial composition. Regression models were conditioned on county economic composition. Data were analyzed in 2016.

**Results:**

Density of retail foods outlets varied greatly by county economic and racial composition; counties with medium-minority populations had the least access to grocery stores and the highest access to fast food restaurants and convenience stores. Low poverty/low-minority population counties had the greatest access to farmer’s markets and grocery stores. For low poverty/low-minority counties, grocery stores were associated with decreased of diabetes prevalence. Supercenters were associated with an increase in diabetes prevalence for high-poverty/low-minority counties. Only low poverty/medium-minority counties had a statistically significant relationship between farmer’s markets and diabetes prevalence. Fast food restaurants were found to be positively associated with diabetes prevalence in all counties except high poverty/medium-minority. However, only low poverty/low-minority counties had a statistically significant relationship. Across all models, access to full service restaurants were significantly associated with lower prevalence of diabetes. Generally, access to convenience stores were associated with increased diabetes prevalence, except for high poverty/low-minority counties.

**Conclusions:**

The food environment is more strongly associated with diabetes prevalence for wealthier counties with a lower proportion of minority residents. This is important given efforts to increase food access in vulnerable communities. Availability of healthier food may not be enough to change health outcomes.

## Background

Type 2 diabetes mellitus (“diabetes”) has increased among adults ages 20 and older in the United States (U.S.) from 5.5 million in 1980 to 1.91.9 million in 2014 [1]. Currently, 11% of U.S. adults have been diagnosed with diabetes, with racial/ethnic minorities being disproportionally affected [1]. Left untreated or poorly managed, diabetes can cause kidney damage, blindness, and vascular insufficiencies leading to lower-limb amputations [2]. Complications from diabetes have led to increasingly high healthcare costs. The total estimated costs associated of diabetes in the United States in 2012 was $245 billion, including $176 billion in direct medical costs and $69 billion in reduced worker productivity [3].

Diabetes risk is influenced by genetics, age, obesity, physical inactivity and poor diets [4]. Consumption of foods with higher amounts of added sugars and fat combined with lower intake of foods with fiber such as fruits, vegetables, and whole grains can increase the risk of diabetes [2]. Dietary behaviors are affected by multiple levels of influence including individual (genetics and personal health beliefs); interpersonal (social networks and supports); community (environmental characteristics); and societal (public policies and systems) [5]. One potential community-level influence on diet which has been the subject of many recent studies is the food environment [6–8]. The food environment is defined as the distribution of food sources within a community, including the number, type, location, and accessibility of retail food outlets [9]. The most common retail food outlets include convenience stores, full-service restaurants and fast-food restaurants.

While some studies have shown that greater access to healthier retail food outlets and lower access to less healthier retail food outlets is associated with more favorable dietary behaviors [6], others have shown conflicting results [10]. In Sallis and Glanz’s (2009) systematic review focusing on the community food environment, the presence of grocery stores or supermarkets in communities increased the probability of having a healthier diet [11]. However, a longitudinal study involving more than 5000 young adults found that having geographic access to more supermarkets was unrelated to fruit and vegetable consumption [12]. Other studies looking at more intermediate health outcomes, such as obesity, have also been mixed [13, 14]. Measuring diet and obesity can be complicated since studies often rely on time-consuming self-reported measures, such as dietary recalls [15, 16]. Additionally, some studies have critiqued the reliability of using BMI as a proxy for weight-related diseases. Since BMI is based on height and weight, it cannot account for differences between fat mass and lean body mass (i.e., muscle). Additionally, BMI threshold categories are based predominately on Caucasians living in Europe and the United States, which can be problematic since some races/ethnicities have higher percentages of body fat, so while they have a normal BMI, they might actually be overweight or obese [17, 18]. Therefore, examining the effect of diet on more proximal, objectively measured disease outcomes, such as diabetes, may provide greater insight into the role of the food environment and health. In general, self-reported diabetes diagnosis has been shown to be a reliable measure [19, 20].

A national study examining retail food outlets and the prevalence of diabetes found that fast food restaurants and convenience stores were positively associated with the prevalence of diabetes and farmer’s markets were negatively associated with them [21]. In another study, county-level data from South Carolina revealed that fast food restaurants were negatively associated with the prevalence of diabetes, yet convenience stores were positively associated with them [22]. In both studies, grocery stores and supercenters were not significantly associated with diabetes prevalence. Mixed findings from these studies could be due to differential effects of the food environment on high and low poverty counties, as well as counties with varying racial/ethnic populations, geographic location, or methodological models used to examine associations. [6–8, 10, 13]. The food environment may play a stronger role in determining the diets and health of lower-income and racial/ethnic minority populations [23, 24]. These populations are more likely to have access to nutrient-deficient foods such as sugar sweetened beverages and salty snacks sold by convenience stores and fast food restaurants and less access to fresh fruits and vegetables sold by larger chain grocery stores or farmer’s markets [25–27]. Yet, the relationship between the retail food environment and health outcomes by socioeconomic status and/or race have not been well documented. One study found that with each additional supermarket in a census tract, fruit and vegetable consumption increased by 32% among African American residents, but only 11% for white residents [6]. Another study focusing on racial disparities in obesity prevalence as explained by the retail food environment found that the food environment explained a greater proportion of obesity prevalence in counties with very high and very low proportions of African American residents [28].

Examining analyses by income and race can help researchers better understand how the relationship between food environment and diabetes prevalence varies for more vulnerable populations. Building a general understanding of this relationship requires research that uses large, national level data. Therefore, this study uses national data to better understand the impact that food environments have on diabetes prevalence. The purpose of this study was to examine the relationship between access to retail food outlets and the prevalence of adult diabetes by county economic and racial composition.

## Methods

### Data

This study uses an existing cross-sectional dataset from the U.S. Department of Agriculture (USDA) Economic Research Service called the Food Environment Atlas (hereafter referred to as *Atlas*) [29]. The *Atlas* is publicly available and contains 2012 data on a number of county-level indicators including: health outcomes, food outlet availability, food access, food insecurity, physical activity levels, and socioeconomic characteristics such as demographic composition; income and poverty levels; county size, and urbanicity. Only counties in the continental U.S. were used in this study (*N* = 3143). Non-gestational diabetes rates in the *Atlas* were obtained from the 2012 CDC’s Behavioral Risk Factor Surveillance System (BRFSS). County-level educational attainment was obtained from the U.S. Census Bureau’s County Population Estimates. After controlling for missing data, the total sample size for this study was 3132 counties. To determine whether data were missing at complete random, we conducted Little’s Missing Completely at Random (MCAR) test [30]. The *p*-value for the test was not significant, suggesting that the data may be assumed to be MCAR. Therefore, we deleted observations with missing values since the number of missing values was not very large. Institutional review board approval was not required for this study because the dataset is publicly available and does not reveal confidential information that can be identified to a particular individual. Data were analyzed in 2016.

### Variables

#### Outcome variables

The outcome variable of interest for this study was the percentage of adults (ages 20 and older with diabetes in a county). Self-reports of medical conditions have higher reliability if the condition is well-defined and relatively easy for an individual to understand [31]. Therefore, to determine an individuals’ diabetic status, BRFSS asked respondents, “Have you ever been told by a doctor that you have diabetes?” If the answer was “yes” they were classified as having diabetes.

#### Explanatory variables

The explanatory variable of interest was the county-level food environment, including the number of (1) grocery stores per 1000 residents, (2) supercenters per 1000 residents, (3) farmer’s markets per 1000 residents, (4) fast food restaurants per 1000 residents, (5) full-service restaurants per 1000 residents, and (6) convenience stores per 1000 residents (see Table 1). Store data are from the U.S. Census Bureau’s County Business Patterns, which reports statistics for nearly 1200 industries based on the 6-digit North American Industry Classification System (NAICS) industry annually. Statistics are available on all business establishments at the U.S. level and by state, county, metropolitan area, zip code, and congressional district levels [32]. Grocery stores were defined as establishments that sell food as their primary business function. Supercenters are defined as establishments that sell food and groceries, as well as merchandise. Farmer’s markets are defined as establishments with at least two vendors selling food products directly to customers. Fast food restaurants are defined as establishments that provide food services in which customers generally order food and pay for before eating. Full-service restaurants are defined as establishments primarily engaged in providing food services to patrons who order and are served while seated (i.e., waiter/waitress service) and pay after eating. Convenience stores are defined as establishments that sell a limited selection of foods.

**Table 1:** Definition of Retail Food Outlets, United States, 2012

Retail Food Outlet	Definition
Grocery stores	Establishments that sell food as their primary business function
Supercenters	Establishments that sell food and groceries, as well as merchandise
Farmer’s markets	Establishments with at least two vendors selling food products directly to customers
Full-service restaurants	Establishments primarily engaged in providing food services to patrons who order and are served while seated (i.e., waiter/waitress service) and pay after eating.
Fast food restaurants	Establishments that provide food to customers that order and pay before leaving
Convenience Stores	Establishments that sell a limited selection of foods

To investigate the association between county economic and racial composition and the prevalence of diabetes, counties were dichotomized based on federal poverty levels. Federal poverty levels are based on the percent of county residents with household income below the poverty threshold. Counties were coded as “high poverty” if their county poverty level was greater than 20% and “low poverty” if their poverty level was less than 20% of the population [27, 28]. We calculated the percent of minority residents for all counties by summing the percent of African American, Hispanic, Asian, American Indian or Alaskan Native, Hawaiian or Pacific Islanders in a county. Using this definition of minority residents, counties were then categorized as low (< 4.6%), medium (4.6%–31.0%), and high (> 31.0%) percent minority residents. These cutoffs represent the 25th and 75th percentiles of the percent minority resident composition of counties [33, 34].

We hypothesized that the association between retail food outlets (grocery stores, supermarkets, farmer’s markets, full-service restaurants, fast food restaurants, and convenience stores) and the prevalence of diabetes would vary by county racial composition. Therefore, interaction terms between racial composition (low, medium, and high percent minority residents) and food outlet type were included in the regression models to test the study’s hypothesis.

#### Other co-variates

The following multiple county-level characteristics were controlled for: number of recreational facilities per 1000 county residents; urbanicity (metro or non-metro); percent of population with a high school degree; and county population size. The number recreational facilities in a county was calculated using the U.S. Census Bureau’s County Business patterns. Recreational facilities included those with the NAICS code of 713,940 and establishments that are primarily engaged in operating fitness and recreational sports facilities [32]. Urbancity of the county was classified by the Office of Management and Budget’s metro and non-metro definitions. Metro areas are defined for all urbanized areas regardless of total area population. Outlying counties are also classified as metro if they are economically tied to the central counties, as measured by the share of workers commuting on a daily basis to the central counties. Non-metro counties are outside the boundaries of metro areas and have no cities with 50,000 residents or more [29]. Percent of population with a high school degree was obtained from the U.S. Census Bureau’s Current Population Survey. County population size was calculated by using data from the 2010 Census of Population and Housing, and includes the total number of individuals residing in a tract which are aggregated to the county level. We also controlled for percent of population 65 years or older and percent of populations under the age of 18 as research shows individuals under the age of 18 are less likely to have type 2 diabetes whereas over the age 65 are much more likely to have type 2 diabetes. These are the only two age variables available in the Food Environment Atlas.

### Statistical analysis

Multiple linear regression models were used to explore the relationship between retail food outlets and diabetes prevalence, and whether this correlation had differential impacts for counties based on economic and racial composition. The following model was used:

$$ \mathrm{y}={\upbeta}_0+{\upbeta}_1\mathrm{RC}+{\upbeta}_2\mathrm{RFA}\ast \mathrm{RC}+{\upbeta}_3\mathrm{X}{+}_{+}\upvarepsilon $$

Where, the prevalence of diabetes is represented by *y* for the county; RC is the county’s racial composition (low-minority population, medium-minority population, high-minority population); RFA is the retail food outlet (grocery store, supercenter, farmer’s market, full-service restaurant, fast food restaurant, and convenience store) per 1000 residents; RFA*RC is an interaction term between RFA and RC for each county, and X is a vector that includes county demographic variables. Regression models were stratified by poverty status (low or high), while controlling for county demographics. The county demographics we controlled for are the number of recreational facilities per 1000 county residents; urbanicity; percent of population with a high school degree; county population size; percent of population 65 years or older; and percent of populations under the age of 18.

Due to the interaction terms between food outlets and minority prevalence (low, medium, and high percent minority residents) in the regression models, we have reported results using average marginal effects. The statistical significance of this effect is tested by the overall cross-partial derivative interaction effects, and their standard errors are estimated the “margins” command in Stata to calculate the average marginal effect of racial composition and retail food outlets. Robust standard errors were used to correct for heterskedasticity. Analyses were performed with the statistical software program Stata: Release 12.

## Results

### County characteristics

The average county-level diabetes prevalence in the U.S. was 10.7%, ranging from a low of 3.3% in Eagle, Colorado to a high of 19.4% in Greene, Alabama (see Table 2). The average number of healthier retail food outlets per 1000 county residents was 0.38 compared to 1.17 less healthier retail food outlets per 1000 county residents. The average percent of non-white residents in a county was 20.1%. A total of 806 counties had a poverty level of 20% or higher and were categorized as “high poverty”; the remaining 2336 counties were categorized as “low poverty”. Among high poverty counties, 123 had low-minority populations; 226 had medium-minority populations, and 456 had high-minority populations. Among low poverty counties, 663 had low-minority populations; 1342 had medium-minority populations, and 328 had high-minority populations.

**Table 2:** County-Level Descriptive Statistics for Study Variables, United States, 2012

Variable	N	Mean (SD)	Min	Max
Health Outcomes				
Adult diabetes prevalence (%)	3138	10.71 (2.25)	3.3	19.4
Food Environment				
Grocery stores/1000 residents	3143	0.26 (0.21)	0	2.99
Supercenters/1000 residents	3143	0.02 (0.22)	0	0.25
Farmer’s markets/1000 residents	3138	0.05 (0.09)	0	1.36
Fast food restaurants/1000 residents	3143	0.58 (0.30)	0	5.8
Full-service restaurants/1000 residents	3143	0.79 (0.59)	0	13.0
Convenience stores/1000 residents	3143	0.60 (0.31)	0	4.13
Physical Activity				
Recreation & fitness facilities/1000 residents	3138	0.07 (0.07)	0	0.77
Socioeconomic Characteristics				
White (%)	3143	78.29 (19.89)	2.67	99.16
Non-white residents (%)	3142	20.11 (19.68)	0.21	97.01
Black (%)	3143	8.75 (14.42)	0	85.44
Hispanic (%)	3143	8.28 (13.19)	0	95.74
Asian (%)	3143	1.14 (2.47)	0	43.01
American Indian or Alaska Native (%)	3143	1.87 (7.61)	0	94.95
Hawaiian or Pacific Islander (%)	3142	0.08 (0.95)	0	48.89
Median household income ($)	3142	43,145 (10,742)	20,577	119,075
Poverty rate (%)	3142	16.76 (6.24)	3.10	50.10
Population 65 years or older (%)	3143	15.88 (4.19)	3.47	43.38
Population under age 18 (%)	3143	23.42 (3.38)	0	41.57
High school degree (%)	3140	34.69 (6.56)	10.9	53.20
County size per 1000 residents	3143	98.23 (312.90)	.082	9818.61
Metro/non-metro counties	3143	0.37 (0.48)	0	1

### Food outlet density analysis by race and income

**Fig. 1:**
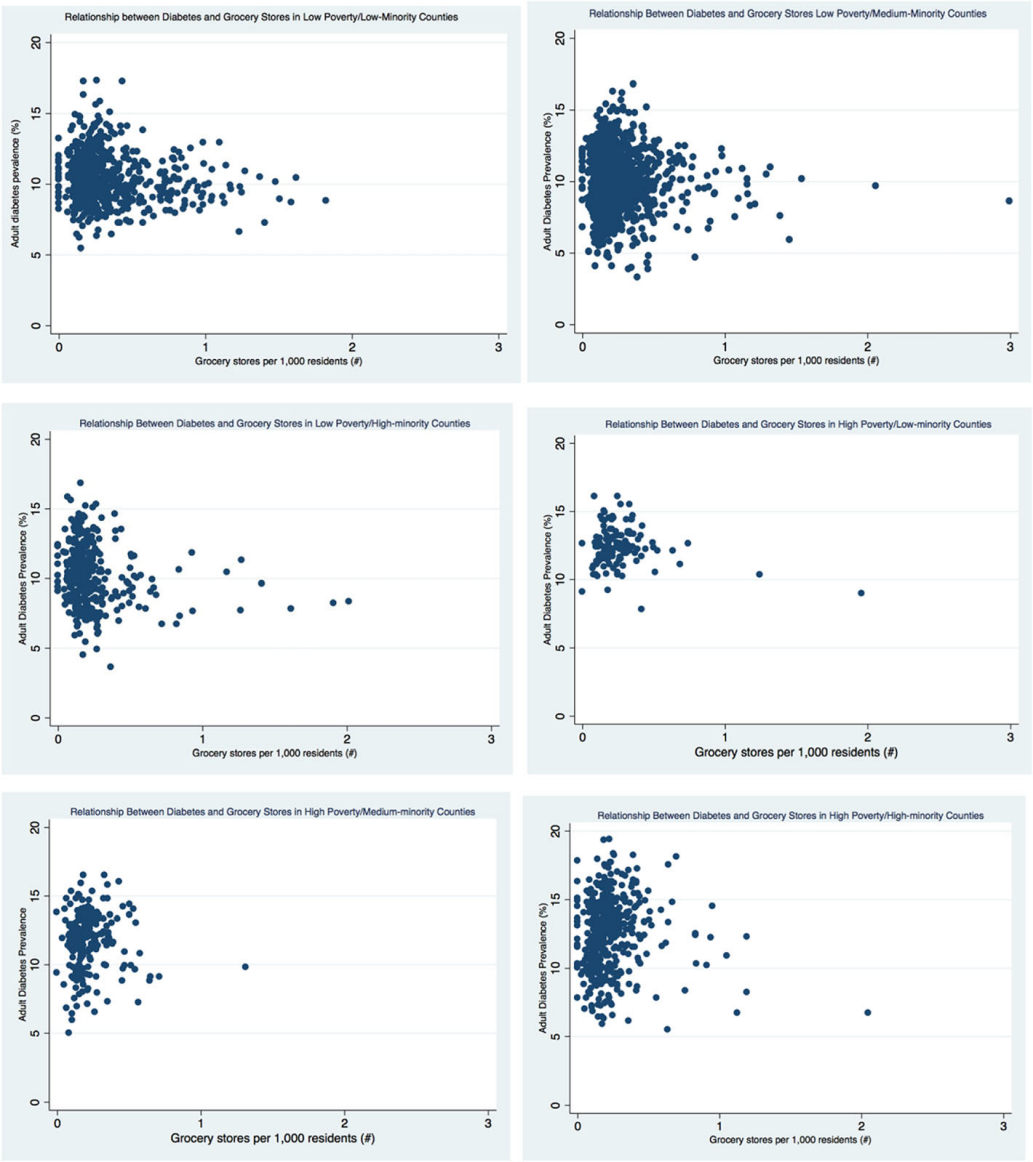
Relationship Between Diabetes and Grocery Stores by County Economic and Racial Composition, 2012

**Fig. 2:**
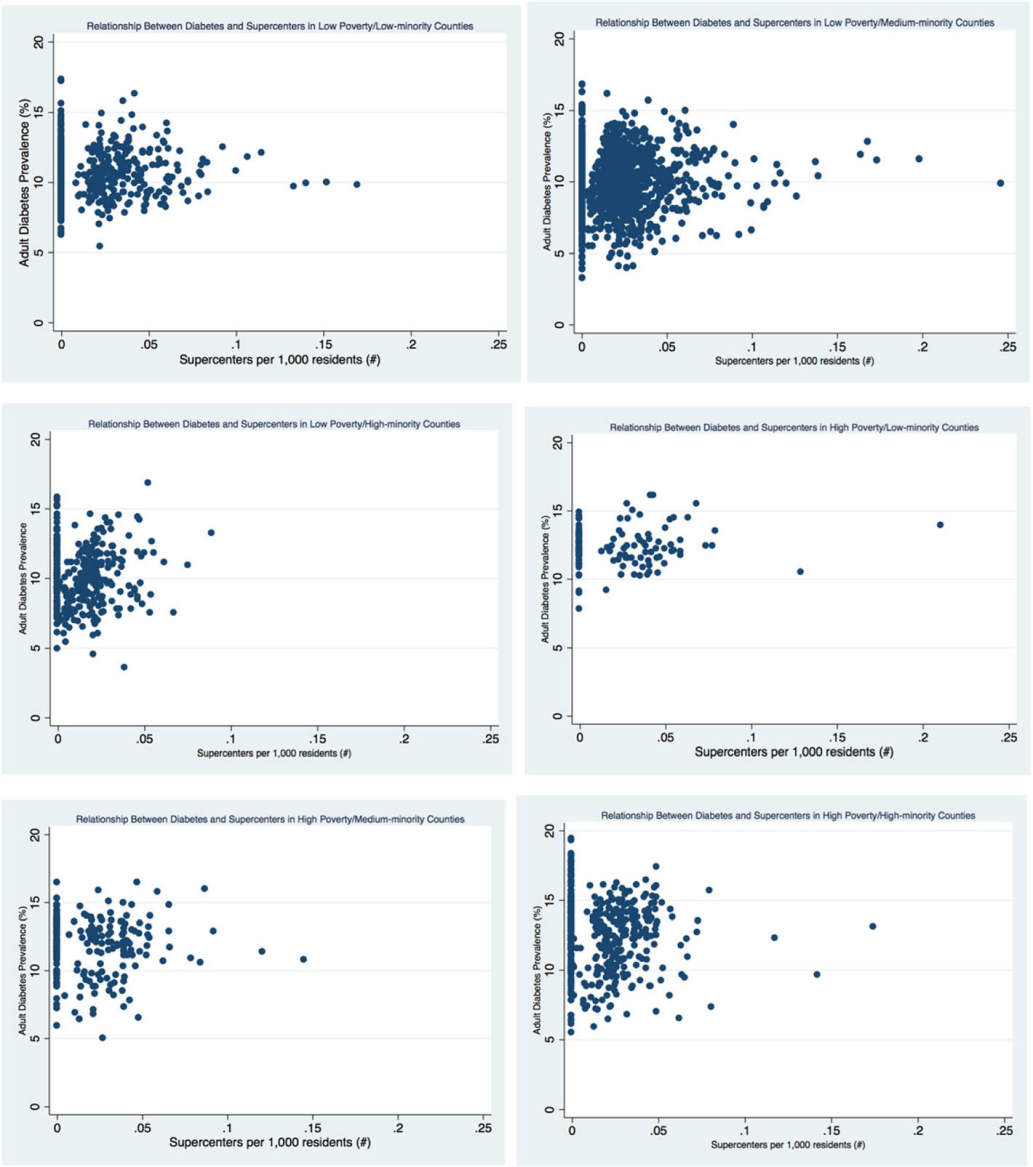
Relationship Between Diabetes and Supercenters by County Economic and Racial Composition, 2012

**Fig. 3:**
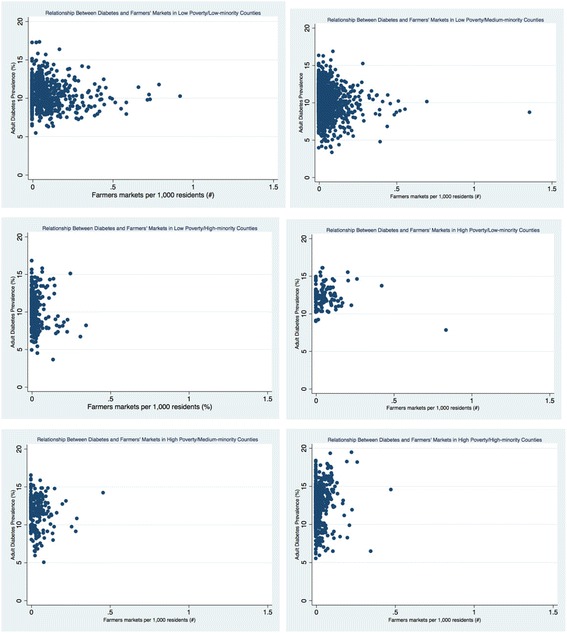
Relationship Between Diabetes and Farmers’ Markets by County Economic and Racial Composition, 2012

**Fig. 4:**
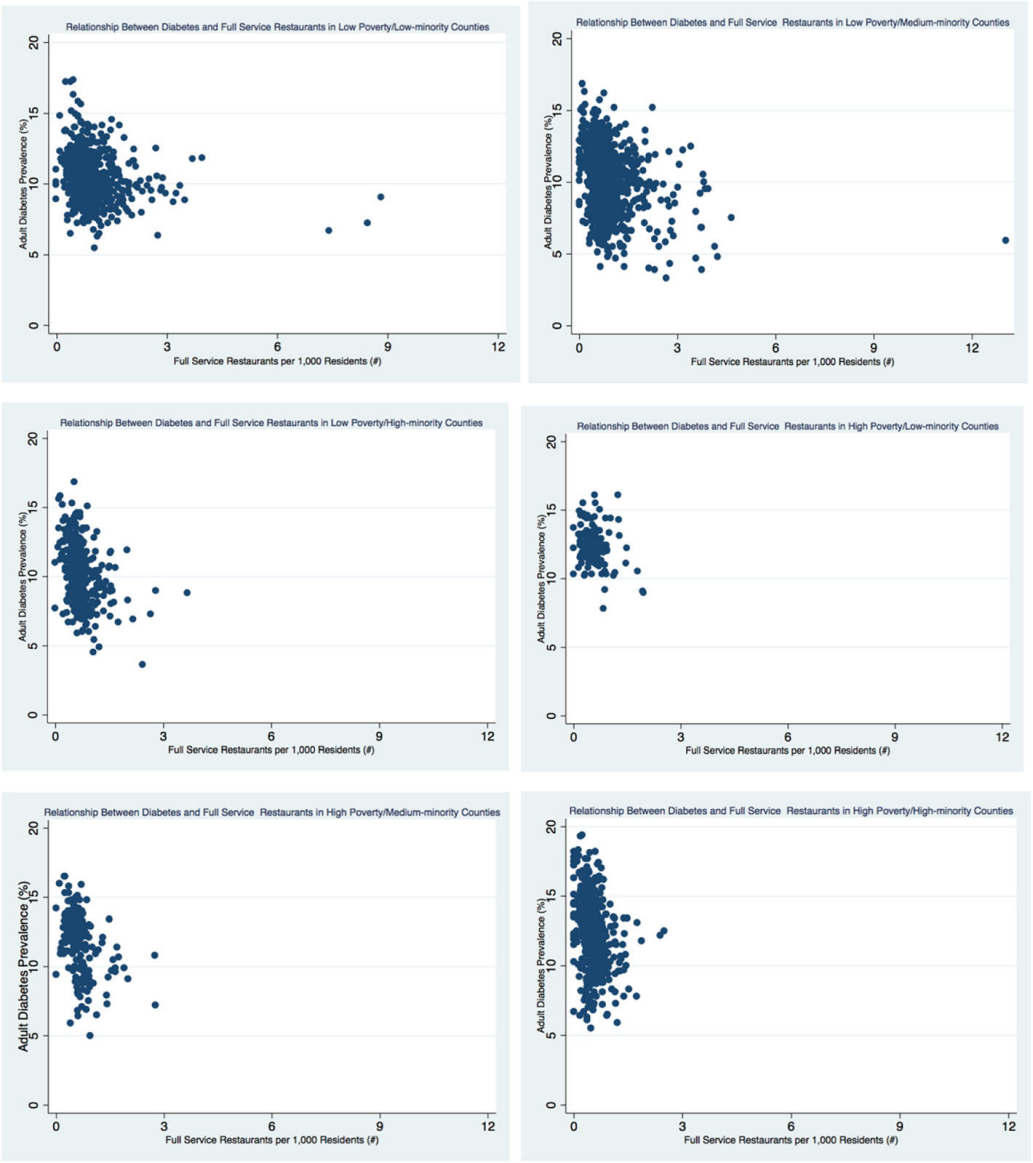
Relationship Between Diabetes and Full Service Restaurants by County Economic and Racial Composition, 2012

**Fig. 5:**
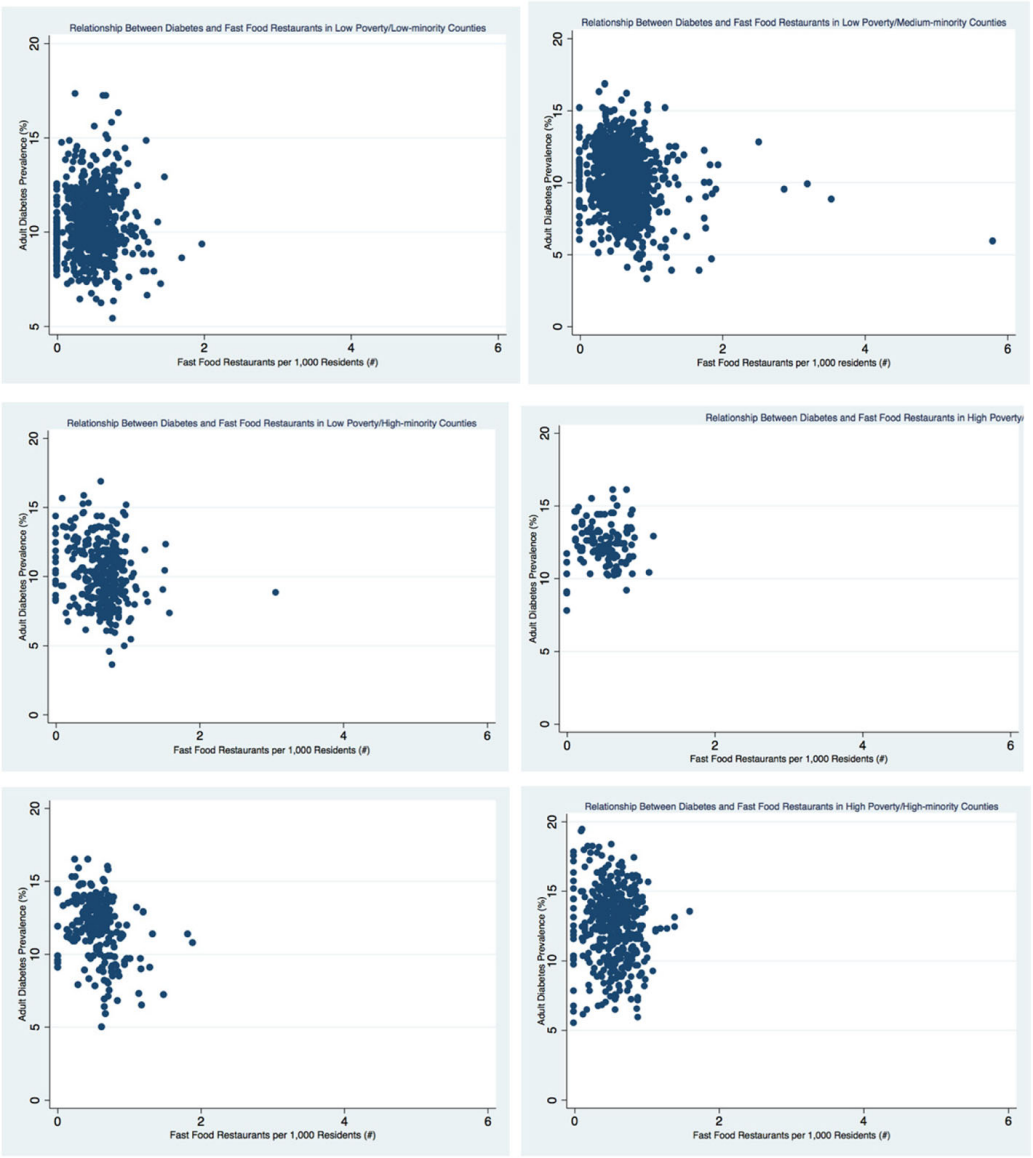
Relationship Between Diabetes and Fast Food Restaurants by County Economic and Racial Composition, 2012

**Fig. 6:**
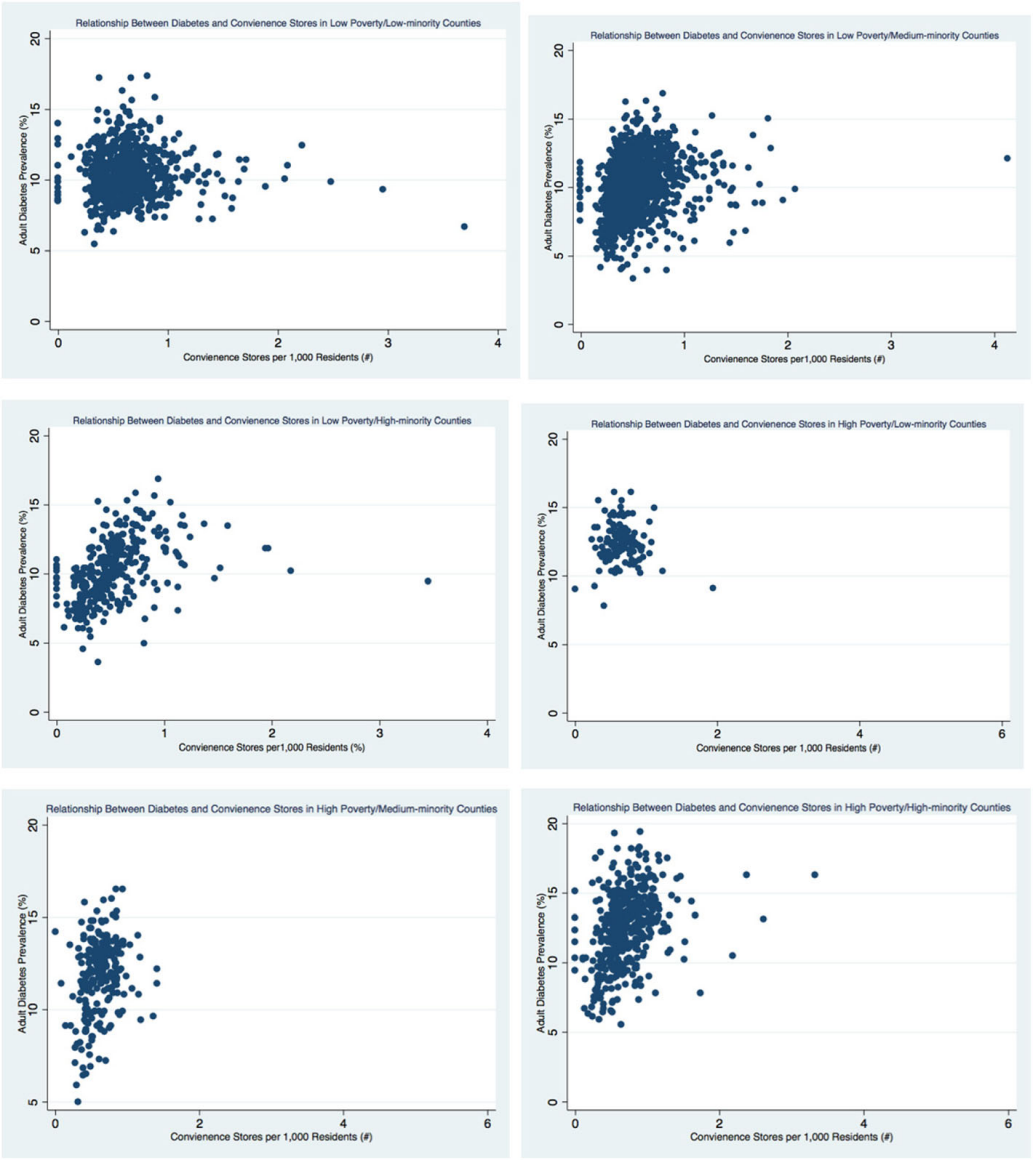
Relationship Between Diabetes and Convenience Stores by County Economic and Racial Composition, 2012

The density of retail foods outlets varied greatly by county economic and racial composition (see Table 3 and Figs. 1, 2, 3, 4, 5, and 6). Among the low and high poverty counties, counties with high and medium-minority populations had the least access to grocery stores (Fig. 1) and the highest access to fast food restaurants (Fig. 5). Low poverty/low-minority population counties had the greatest access to farmer’s markets (0.09 stores per 1000 residents) among all six county types - nearly triple the number of stores than counties with high-minority populations (Fig. 3). High and low poverty counties had similar access to supercenters, regardless of minority level (Fig. 2). High poverty/high-minority counties were shown to have the least access to full-service restaurants (0.54 stores per 1000 residents), almost half the number of full-service restaurants as compared to low poverty/low-poverty counties (0.98 stores per 1000 residents) – see Fig. 4. High poverty/high-minority counties had the greatest access to convenience stores (0.71 stores per 1000 residents) – see Fig. 6.

**Table 3:** Food Outlet Density by County Economic and Racial Composition, United States, 2012

Economic Composition (Poverty Levels)	Racial Composition (% Minority Residents)	*N*	Grocery Stores per 1000 Residents	Super centers per 1000 Residents	Farmers Markets per 1000 Residents	Fast food Restaurants per 1000 Residents	Full-service Restaurants per 1000 Residents	Convenience Stores per 1000 Residents
Low Poverty	Low	663	0.33 (0.27)	0.01 (0.23)	0.09 (0.12)	0.49 (0.28)	0.98 (0.73)	0.65 (0.33)
	Medium	1345	0.23 (0.19)	0.02 (0.24)	0.05 (0.08)	0.62 (0.32)	0.84 (0.63)	0.54 (0.27)
	High	328	0.24 (0.24)	0.01 (0.02)	0.03 (0.05)	0.65 (0.31)	0.73 (0.42)	0.53 (0.35)
High Poverty	Low	123	0.26 (0.22)	0.02 (0.03)	0.06 (0.10)	0.51 (0.25)	0.59 (0.36)	0.66 (0.24)
	Medium	226	0.22 (0.14)	0.02 (0.02)	0.04 (0.07)	0.56 (0.28)	0.65 (0.38)	0.63 (0.22)
	High	456	0.23 (0.19)	0.02 (0.02)	0.03 (0.05)	0.55 (0.26)	0.54 (0.33)	0.71 (0.34)

*Notes*: Standard deviations listed in parenthesis

### Regression analyses

Table 4 shows OLS regression results for diabetes prevalence by county economic and racial composition for the six models. For low poverty/low-minority counties, each additional grocery store per 1000 residents was associated with 0.67 percentage point decrease in the prevalence of diabetes (*p* < .001). Supercenters were associated with a statistically significant (*p* < .10) increase in the prevalence of diabetes for high-poverty/low-minority counties. Each additional supercenter per 1000 residents was associated with a 5.36 percentage point increase in the prevalence of diabetes. When examining access to farmer’s markets in low poverty counties, greater access to farmer’s markets had a larger effect on decreased diabetes prevalence in medium and high-minority population counties than counties with low-minority populations. However, only the low poverty/medium-minority counties had a statistically significant relationship between farmer’s markets and diabetes prevalence.

**Table 4:** Marginal Effect of Food Outlets on Diabetes Rates by County Economic and Racial Composition, United States, 2012

Economic Composition (Poverty Levels)	Racial Composition (% Minority Residents)	Grocery Stores per 1000 Residents	Supercenters per 1000 Residents	Farmers Markets per 1000 Residents	Fast food Restaurants per 1000 Residents	Full-service Restaurants per 1000 Residents	Convenience Stores per 1000 Residents
Low Poverty *N* = 2329	Low	−0.67 (−1.12, −0.22) 0.004	2.94 (−1.66, 7.54) 0.212	−0.51 (−1.34, 0.32) 0.233	0.64 (0.17, 1.10) 0.007	−0.49 (−0.74, −0.24) 0.000	0.31 (−0.06, 0.68) 0.103
	Medium	−0.19 (−0.77, 0.38) 0.512	−2.68 (−6.35, 0.98) 0.151	−2.50 (−3.89, −1.11) 0.000	0.30 (−0.16, 0.76) 0.21131	−0.69 (−0.93, −0.45) 0.000	1.57 (1.21, 1.93) 0.000
	High	0.73 (−0.32, 1.80) 0.171	0.25 (−14.78, 15.29) 0.971	−2.33 (−8.80, 4.13) 0.483	0.63 (−0.32, 1.58) 0.201	−1.72 (−2.42, −1.01) 0.000	1.68 (0.90, 2.45) 0.000
High Poverty *N* = 803	Low	−0.47 (−1.61, 0.67) 0.422	5.36* (−0.53, 11.24) 0.074	−1.99 (−6.43, 2.44) 0.383	0.29 (−0.80, 1.38) 0.612	−1.37 (−2.15, −0.59) 0.001	−0.33 (−1.27, 0.62) 0.501
	Medium	−0.14 (−1.96, 1.93) 0.992	4.42 (−5.90, 14.74) 0.401	1.53 (−1.82, 4.89) 0.373	−0.45 (−1.50, 0.59) 0.392	−2.00 (−2.75, −1.26) 0.001	1.63 (0.46, 2.80) 0.006
	High	0.46 (−1.02, 1.95) 0.542	−4.13 (−14.03, 5.77) 0.414	2.80 (−4.35, 9.96) 0.444	0.06 (−0.98, 1.09) 0.921	−2.25 (−3.14, −1.36) 0.002	1.88 (1.17, 2.59) 0.001

*Notes:* 95% confidence intervals are listed in brackets. *P*-values are listed below confidence intervals and are two-sided. OLS Regression models controlled for number of recreational facilities per 1000 county residents; percent of population with a high school degree; county population size; urbancity; percent of population 65 years or older; and percent of populations under the age of 18

Fast food restaurants were found to be positively associated with the prevalence of diabetes in all communities except high poverty/medium-minority. However, only low poverty/low-minority counties had a statistically significant relationship (*p* < .001). Each additional fast food restaurant per 1000 residents was associated with a 0.64 percentage point increase in diabetes prevalence.

Across all models, access to full service restaurants were significantly associated with lower prevalence of diabetes. High poverty/high-minority population counties had a stronger association with access to full service restaurants and diabetes rates, with each additional full-service restaurant per 1000 residents being associated with a 2.25 percentage point decrease in the prevalence of diabetes (*p* < .001), compared to a 1.72 percentage point decrease (*p* < .001) for low poverty/high-minority population counties and a 0.69 percentage point decrease for low poverty/medium-minority population counties (*p* < .001). Generally, access to convenience stores were associated with increased diabetes prevalence, except for high poverty/low-minority counties. For high poverty/high-minority counties each additional convenience store per 1000 residents was associated with 1.88 percentage point increase in diabetes (*p* < .001) prevalence compared to only a 0.31 percentage point increase in low poverty/low-minority counties (*p* < .10).

## Discussion

This study examined the relationship between access to retail food outlets and the prevalence of adult diabetes by county economic and racial composition. Surprisingly, full-service restaurants were associated with decreased diabetes prevalence regardless of income or race. This may be due in part to high poverty counties having less access to full-service restaurants, which might be more expensive to eat at, therefore reducing their ability to purchase meals there. Additionally, full-service restaurants in low poverty counties may be more likely to serve healthier, upscale meals. One study found that a higher density of full-service restaurants was associated with lower weight status. The authors hypothesized that customers sought seeking healthier foods might be more likely to eat at full-service restaurants [35].

Consistent with other research, convenience stores were associated with higher diabetes rates in high and low poverty counties across all racial compositions except for high poverty/low-minority areas. High minority counties had the least access to grocery stores and farmer’s markets (regardless of poverty level). Access to farmer’s markets was only associated with decreased diabetes rates in low poverty/low-minority counties – even though they only had the second highest density farmers’ markets. Only low poverty/low-minority counties benefited from having a higher density of grocery stores. This may be due to these counties having greater access to grocery stores than any other county type.

One possible explanation for the lack of statistical significance in this study among high poverty counties is that at lower income levels, availability of healthy food makes little difference since it is still unaffordable for many [36, 37]. Conversely, wealthier counties with disposable income may have more opportunities to spend money on food. While previous studies have found supermarkets and grocery stores to be negatively associated with county-level diabetes rates, they did not look at the interaction between race and poverty [24, 38]. One study that did examine race found there was a greater positive correlation between grocery stores and fruit and vegetable among African American residents than white residents [6]. While we did not find similar positive benefits for access to grocery stores among high-minority counties, this may signal that including the association of both race and poverty at the county-level may better reflect the effects of retail food outlets on health than just race or poverty status alone [35].

Consistent with previous research, this study found that high poverty and high-minority areas had lower access to grocery stores and farmer’s markets [24, 27]. Bower and colleagues (2014) found that high poverty communities had fewer supermarkets regardless of race/ethnicity, but at equal levels of poverty, predominantly African American had the fewest supermarkets [27]. Furthermore, lower socioeconomic communities had greater access to convenience stores and fast food restaurants than higher socioeconomic communities [10, 13]. For this study, high poverty/high-minority populations had the greatest access to convenience stores and fast food restaurants and the least access to grocery stores and farmer’s markets among any other county type. Conversely, counties with low poverty/low-minority populations had the greatest access to grocery stores and farmer’s markets than any other county type – nearly triple the density of farmer’s markets than counties with high poverty/high-minority populations.

### Study limitations

Like all studies, this study has limitations. This study is cross-sectional in nature and therefore cannot make conclusions about causality. To explore the potential relationship between diabetes prevalence and the food environment based on racial and economic differences we stratified regression models based on these county variables. Since we created binary poverty and categorical racial/ethnical variables based on continuous variables, this may add to the complexity and interpretation of our results and increases the chance for loss of information. Additionally, the county-level diabetes rates in this study are based on BRFSS and they do not differentiate between Type 1 and Type 2 diabetes. While approximately 90–95% of diabetes cases in the U.S. are Type 2, the associations we found are not exclusively based on Type 2 [3]. Diabetes is self-reported in BRFSS, and while studies have shown that self-reported diabetes is fairly reliable measure of diagnosis, there is still room for under- or over- reporting by individuals. Another limitation about BRFSS is that it obtains county-level estimates by aggregating 3 years of data for a single estimate instead of a single year. The ecological nature of this study and the research-imposed grouping of counties by income and race may over-generalize county associations. We did control for urbanicity, percent of population with a high school degree, and county population size to help address this limitation.

This study cannot determine whether the effect seen on county diabetes rates using type of retail food outlets is due specifically to access to food outlets or to other factors not measured in the model. There are many other factors that affect diabetes rates, including genetics, personal health beliefs or food preferences. Lastly, this study did not control for the possibility that individuals might access food outlets in neighboring counties because they live close to county borders.

## Conclusion

This study investigated the relationship between access to retail food outlets and diabetes prevalence by county economic and racial composition. Overall, our results suggest that the food environment is central when examining diabetes prevalence, specifically in wealthier counties with a low proportion of minority residents. Some studies have begun to focus on innovative methods for addressing healthy food access in disadvantaged neighborhoods including building supermarkets [39], hosting mobile farmer’s markets [40], and assisting smaller food stores to carry healthier food items [41]. More studies are needed to examine these relationships, and if possible, future studies should focus on longitudinal study designs so researchers can track individuals over time. However, this study’s finding is still particularly important given recent efforts nationwide to increase food access in lower-income and minority communities. These efforts may not effectively improve disease rates if they cannot address other food access issues such as cost of healthy food, quality of healthy food (i.e., fruits and vegetables) and education to help low-income individuals learn how to prepare and cook with healthier foods [26]. Public health researchers and policymakers should recognize that increasing availability of healthier food outlets may not be enough to change diet and health outcomes.
